# Exciton emission of quasi-2D InGaN in GaN matrix grown by molecular beam epitaxy

**DOI:** 10.1038/srep46420

**Published:** 2017-04-18

**Authors:** Dingyu Ma, Xin Rong, Xiantong Zheng, Weiying Wang, Ping Wang, Tobias Schulz, Martin Albrecht, Sebastian Metzner, Mathias Müller, Olga August, Frank Bertram, Jürgen Christen, Peng Jin, Mo Li, Jian Zhang, Xuelin Yang, Fujun Xu, Zhixin Qin, Weikun Ge, Bo Shen, Xinqiang Wang

**Affiliations:** 1 State Key Laboratory of Artificial Microstructure and Mesoscopic Physics, School of Physics, Peking University, Beijing 100871, China; 2 Collaborative Innovation Center of Quantum Matter, Beijing 100871, China; 3 Leibniz Institute for Crystal Growth, Berlin 12489, Germany; 4 Institute of Experimental Physics, Otto-von-Guericke-University Magdeburg, Universitätsplatz 2, Magdeburg 39106, Germany; 5 Key Laboratory of Semiconductor Materials Science and Beijing Key Laboratory of Low-dimensional Semiconductor Materials and Devices, Institute of Semiconductors, Beijing, CAS, 100083, China; 6 Microsystem & Terahertz Research Center, 596 Yinhe Road, Shuangliu, Chengdu 610200, China

## Abstract

We investigate the emission from confined excitons in the structure of a single-monolayer-thick quasi-two-dimensional (quasi-2D) In_x_Ga_1−x_N layer inserted in GaN matrix. This quasi-2D InGaN layer was successfully achieved by molecular beam epitaxy (MBE), and an excellent in-plane uniformity in this layer was confirmed by cathodoluminescence mapping study. The carrier dynamics have also been investigated by time-resolved and excitation-power-dependent photoluminescence, proving that the recombination occurs via confined excitons within the ultrathin quasi-2D InGaN layer even at high temperature up to ~220 K due to the enhanced exciton binding energy. This work indicates that such structure affords an interesting opportunity for developing high-performance photonic devices.

In recent years, a series of two-dimensional (2D) materials, such as graphene, transition metal dichalcogenides and black phosphorus, have attracted much research attention due to their remarkable physical properties and novel applications^1^. However, the optoelectronic devices based on these materials are mostly limited by several difficulties, including fabrication of large-area high-quality materials, making high efficient doping, subsequent Ohmic contact, and so on^2^. This encourages people to search for new approaches and materials which should not only show novel 2D nature in ultrathin layers but are also suitable for bulk planar technology. Atomically thick quasi-2D III-nitride is a promising candidate which is experimentally available and hence hopeful to enlarge the family of 2D materials, leading to emerging applications in optoelectronic devices^3^,^4^.

In_x_Ga_1−x_N is widely used as an active layer and well applied in the fields of light emitting diodes, laser diodes, solar cells and photoelectrochemical water splitting devices^5^,^6^,^7^. That benefits from the wide and tunable bandgap of InGaN material from the infrared (InN at 0.64 eV) to ultraviolet (GaN at 3.4 eV) region, covering the entire visible spectrum with perfect match to the solar spectrum and making it irreplaceable by a similar GaAs based quantum structure^8^,^9^. However, fabrication of high quality thick InGaN films on GaN suffers from two obstacles, i.e. high density threading dislocations and phase separation with In-rich clusters. The former one arises from large thermal/lattice mismatch between InN and GaN template, while the latter one results from very low InN solubility in GaN at common growth temperature^10^,^11^. A promising solution to avoid the two obstacles is to use pseudomorphic growth. That is exactly what the proposed atomically thick quasi-2D III-nitride in this letter can satisfy, where the quasi-2D In(Ga)N is coherently grown on GaN barrier^12^,^13^,^14^,^15^. The confined carriers in this quasi-2D InGaN may thus lead to a high emission efficiency. Unfortunately, the carrier dynamics in such ultrathin InGaN layer are not well investigated to the best of our knowledge, although we notice that the carrier dynamics in thicker (~2–6 nm) InGaN have been studied previously^16^,^17^,^18^,^19^,^20^. In this letter, the carrier dynamics in such quasi-2D InGaN inserted in GaN matrix have been studied for the first time. The optical properties were investigated by temperature-dependent, time-resolved and excitation-power-dependent photoluminescence (PL), confirming that the PL emission does originate from the recombination of confined excitons for temperatures up to ~220 K.

## Results

**Figure 1:**
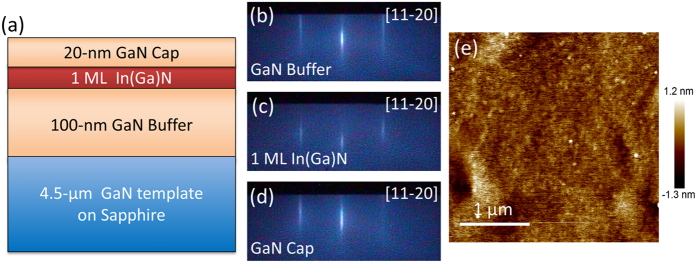
(**a**) Schematic image of the sample structure of the quasi-2D InGaN layer inserted in GaN matrix. Typical RHEED patterns after growth of the GaN buffer layer (**b**), single ML In(Ga)N (**c**) and GaN cap layer (**d**), respectively. (**e**) Typical AFM image of GaN cap layer.

First, we designed the sample structure as shown in Fig. 1(a), consisting of 1 monolayer (ML) In(Ga)N inserted in a GaN matrix. The reason we use In(Ga)N here is that we only deposit 1 ML InN although, as we will show later, it is actually InGaN layer. The sample was grown by plasma-assisted molecular beam epitaxy (MBE, SVTA) and the growth was *in-situ* monitored by reflection high-energy electron diffraction (RHEED). A 4.5 μm-thick GaN layer on *c*-plane sapphire was used as the template. After thermal cleaning at 600 °C for 30 mins, 100 nm-thick GaN layer was grown at 810 °C under Ga-rich condition. Then, an annealing step was carried out to completely eliminate the Ga adatoms, followed by the deposition of InN with a coverage of 1 ML under slightly In-rich conditions at 600 °C. Finally a 20 nm-thick GaN cap layer was grown at the same temperature. The growth rate of the epitaxial layers was 0.7 ML/sec and the whole epi-layer was undoped. The growth temperature of InN used here is about 100 °C higher than that commonly adopted for thick InN films. Fig. 1(b–d) show the RHEED pattern images recorded at the end of each layer. As shown in Fig. 1(b), the 2D growth mode for the GaN buffer layer is confirmed by the streaky RHEED pattern. The brightness of the RHEED after annealing step was almost the same as that for the initial GaN template, proving that no excess Ga adatoms were left over. For deposition of the single ML InGaN, the RHEED pattern kept almost the same as above except that the intensity is slightly weaker, as shown in Fig. 1(c). The spacing between the diffraction streaks didn’t change, indicating that the quasi-2D InGaN layer was coherently grown on the GaN barrier. The RHEED pattern was kept streaky during the growth of the GaN cap layer as shown in Fig. 1(d). The surface morphology of the GaN cap layer was then investigated by atomic force microscopy (AFM) as shown in Fig. 1(e), with a surface roughness (root mean square, RMS) of 0.34 nm for a typical scanned area of 3 × 3 μm^2^.

**Figure 2:**
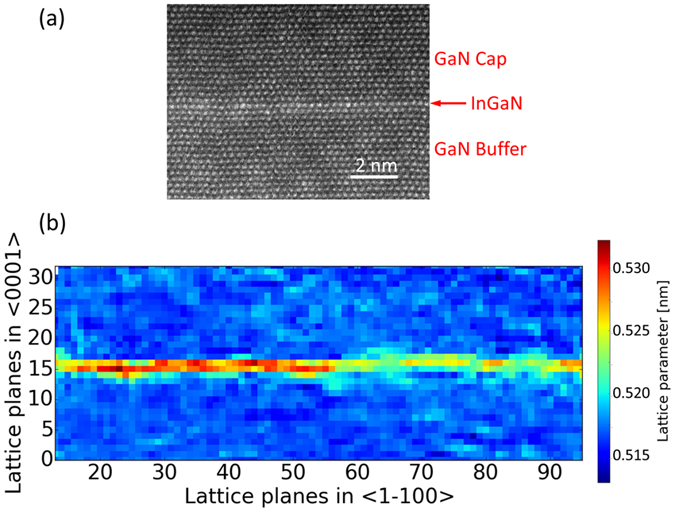
(**a**) High-resolution STEM image for the sample. The red arrow marks the position of single ML InGaN. (**b**) Map of the *c*-lattice parameter in the region of the quasi-2D InGaN layer.

To confirm the successful growth of the quasi-2D InGaN layer, cross sectional scanning transmission electron microscopy (STEM) was performed as displayed in Fig. 2(a). One-ML-thick quasi-2D layer marked with red arrow can be recognized in the image, which appears brighter compared to the surrounding GaN due to the higher atomic number of the indium atom. No misfit dislocations were found at the InGaN/GaN interface, confirming a coherent growth of the quasi-2D InGaN layer. To determine the composition of the InGaN layer, a map of *c*-lattice parameter was measured as shown in Fig. 2(b), according to the method described previously^21^. In contrast to Fig. 2(a), Fig. 2(b) show almost the same area but expanded. We have compared the mean lattice parameter in the InGaN region against supercell simulations using an empirical potential (see ref. 14 for details). With respect to GaN, the strain yields 2.1%, which yields an indium content of the InGaN layer being around 25% on average. Thus, our structural analyses confirm that the quasi-2D layer is an InGaN rather than pure InN despite that we did deposit InN itself. That result probably comes from the relatively high growth temperature of the InN layer, which enhances not only the decomposition rate of InN but also the possibility of atom inter-diffusion. More work is needed to clarify the origin and the growth technique should be further developed to precisely control the indium composition in this quasi-2D layer.

**Figure 3:**
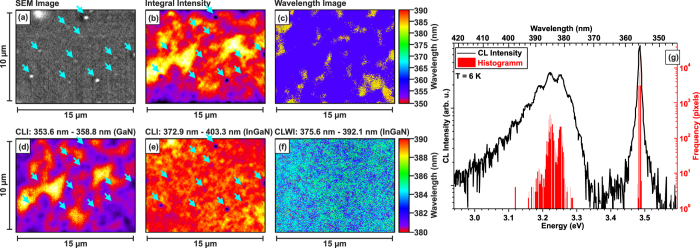
(**a**) SEM image of the investigated sample surface. The cyan-blue arrows mark some In-rich droplets. The integral intensity CL image (**b**) reveals reduced intensity at the droplet positions. (**c**) distribution of the CL peak wavelength and corresponding monochromatic CL images in the spectral region of (**d**) GaN matrix and (**e**) quasi-2D InGaN layer. The monochromatic images are scaled to their individual minima and maxima intensities. (**f**) CL wavelength image of the InGaN luminescence contribution, exclusively. (**g**) spatially integrated CL spectrum of the area shown in SEM image (**a**) as well as the histogram of the wavelength image shown in (**c**).

Highly spatially and spectrally resolved cathodoluminescence (CL) measurements at 6 K have been performed to investigate the lateral uniformity and emission properties of the quasi-2D InGaN layer^22^. In Fig. 3(a), a plan-view scanning electron microscopy (SEM) image of the sample is depicted, in which a smooth, mirror-like surface is visible with some small In-rich droplets marked by cyan-blue arrows. The CL intensity at the droplet position is reduced due to shadowing of the emission, as revealed by the spectrally integral CL intensity image shown in Fig. 3(b). Figure 3(g) shows the CL spectrum measured at 6 K. The luminescence is dominated by a near-band-edge (NBE) emission of GaN (peak wavelength of 355.7 nm) as well as the broad emission of the quasi-2D InGaN layer appearing around 385 nm. The monochromatic intensity images Fig. 3(d) show a rather inhomogeneous intensity distribution in case of the GaN NBE peak, in complete contrast to the quasi-2D InGaN emission which exhibits a relatively homogeneous distribution of the CL intensity (Fig. 3(e)) as well as peak wavelength (Fig. 3(f)). We found a local switching between Fabry-Pérot-Modes for the InGaN emission which leads to the observed modulated integral spectrum. To statistically analyze the fluctuations of the GaN and quasi-2D InGaN luminescence, we calculated the histogram of the wavelength image, where the frequentness, i.e. the number of pixels in the CL wavelength map emitting at certain photon energy, is plotted versus photon energy for all 51200 pixels as shown in Fig. 3(g). Two obvious features can be seen from Fig. 3: (1) The emission from the quasi-2D InGaN layer is intense, which reveals the luminescence resulting from strong carrier confinement in the quasi-2D layer; (2) The emission from the quasi-2D InGaN as shown in Fig. 3(e,f) is quite uniform in terms of intensity and wavelength and thus the CL mapping is a strong indication of the excellent in-plane uniformity of the quasi-2D InGaN layer.

**Figure 4:**
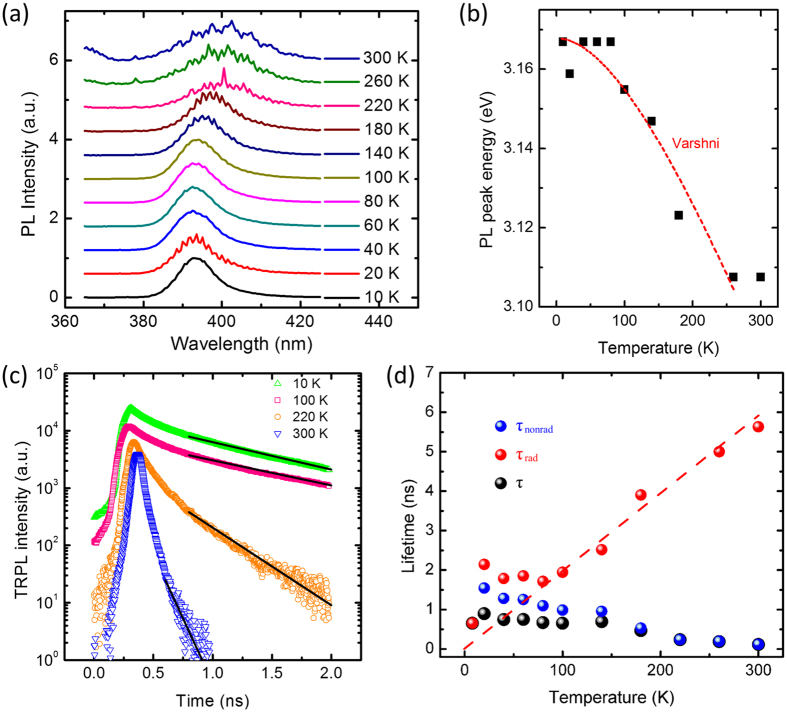
(**a**) Normalized temperature-dependent PL spectra. (**b**) PL peak energy at different sample temperatures and the corresponding best fitting line by the Varshni model. (**c**) TRPL transients and the PL lifetimes by fitting lines (black solid lines). (**d**) The evolution of PL lifetimes (black dots), radiative lifetimes (red dots) and non-radiative lifetimes (blue dots) versus the sample temperatures. The best fitting line (red dashed line) shows the linear nature for radiative lifetimes.

Turning to the carrier dynamics, we measured temperature-dependent PL spectra, as can be seen in Fig. 4(a). The emission around 395 nm is originating from quasi-2D InGaN layer. The difference of the peak emission between PL and CL may originate from a slight non-uniformity of the indium composition due to the wafer temperature fluctuation. The temperature-dependent emission peak wavelength is plotted in Fig. 4(b) and it shows that the peak wavelength redshifts from 392 nm to 399 nm with temperature increasing from 10 K to room temperature (RT). This redshift comes from temperature dependence of the energy gap, following Varshni model 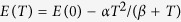
23. The red dashed line in Fig. 4(b) is the bestfit line by the Varshni model with parameters of *E*(0) = 3.168 eV, *α* = 0.5 meV K^−1^ and *β* = 290 K. The results are reasonable in comparison with the previously reported ones for InGaN or GaN materials^24^,^25^. The typical time-resolved PL (TRPL) transients are shown in Fig. 4(c). The signals can be characterized by biexponential decay curves and the slower decay time is fitted by a black solid line and taken to represent the PL lifetime *τ*^26^. We assume the non-radiative recombination process is frozen out and thus neglected at 10 K^27^. The evolution of the radiative and non-radiative recombination lifetimes versus the sample temperatures can then be calculated from the temperature behaviors of both the integrated PL intensity and PL lifetime, as summarized in Fig. 4(d)^28^. At temperatures above 80 K, the radiative lifetime *τ*_rad_ (red dots) increases linearly with a slope of 19.7 ps K^−1^. This linear nature is a typical feature of the confinement within quasi-2D InGaN layer^29^.

## Discussion

**Figure 5:**
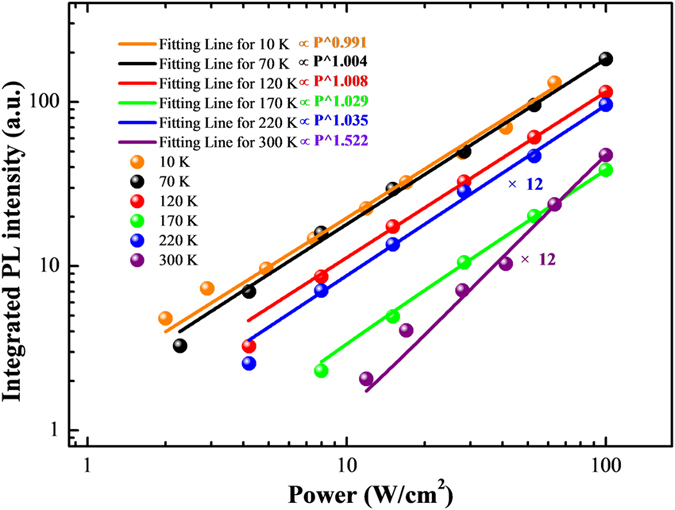
Dependence of the integrated PL intensity on the excitation power. The PL intensity for 220 K or 300 K was multiplied by 12 times.

The origin of the light emission from such quasi-2D InGaN layer is not clear since the exciton binding energy for bulk InN is numerically predicted as only about 6.1 meV^30^. As shown in Fig. 5, we performed the excitation-power-dependent PL, to further clarify whether the emission around 395 nm originates from recombination of excitons or photo-generated free carriers. It is believed the integrated PL intensity increases with excitation power density as the relation 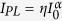, where 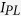 is the integrated PL intensity, *I*_0_ is the excitation power density and *η* is related to the PL efficiency. The exponent *α* depends on the recombination mechanism and is expected to be close to 1 for free exciton and around 2 for free carrier^31^,^32^. As shown in Fig. 5, the parameter *α* is around 1 at temperatures up to ~220 K, while it is ~1.522 at RT. This reveals that the emission in quasi-2D InGaN almost completely originates from recombination of excitons up to ~220 K and partially originates from free carriers at RT. This excitonic nature of the PL emission from the quasi-2D InGaN layer can be ascribed to the enhanced exciton binding energy in the quasi-2D InGaN confined layer.

In summary, we present in this letter the fabrication and exciton emission of quasi-2D InGaN layer inserted in GaN matrix. The STEM study confirms the successful growth of the quasi-2D InGaN layer. Strong emission is obtained from the quasi-2D layer by CL measurement. The radiative lifetime increases linearly with sample temperature, showing a typical feature of the confinement. The exciton emission is further clarified by excitation-power-dependent PL spectra. This proposed novel quasi-2D InGaN affords possibility for developing high-performance photonic devices, as its avoidable generation of misfit dislocations and enhanced carrier confinement.

## Methods

### Characterizations

We characterized our sample by the measurements of STEM, SEM, CL, PL and AFM. STEM was performed using a FEI Titan 80–300 kV electron microscopy operated at 300 keV. CL investigations were carried out using a custom-build system based on an SEM JEOL JSM 6400 equipped with a monochromator and an intensified Si-diode array. For TRPL measurements, a streak camera (OPTRONIS SC101) was used as the detector, and the sample was excited by a Ti:sapphire fs laser with an excitation wavelength of 237 nm and an excitation power density of ~50 W/cm^2^. The surface morphology was characterized by Bruker Dimension ICON-PT AFM.

## Additional Information

**How to cite this article**: Ma, D. *et al*. Exciton emission of quasi-2D InGaN in GaN matrix grown by molecular beam epitaxy. *Sci. Rep.* **7**, 46420; doi: 10.1038/srep46420 (2017).

**Publisher's note:** Springer Nature remains neutral with regard to jurisdictional claims in published maps and institutional affiliations.
